# Supplementary figures and images for: Toxoplasma gondii excretion of glycolytic products is associated with acidification of the parasitophorous vacuole during parasite egress
Source: PLoS Pathog. 2022 May 5;18(5):e1010139. doi: 10.1371/journal.ppat.1010139 (PMC9113570; doi:10.1371/journal.ppat.1010139)

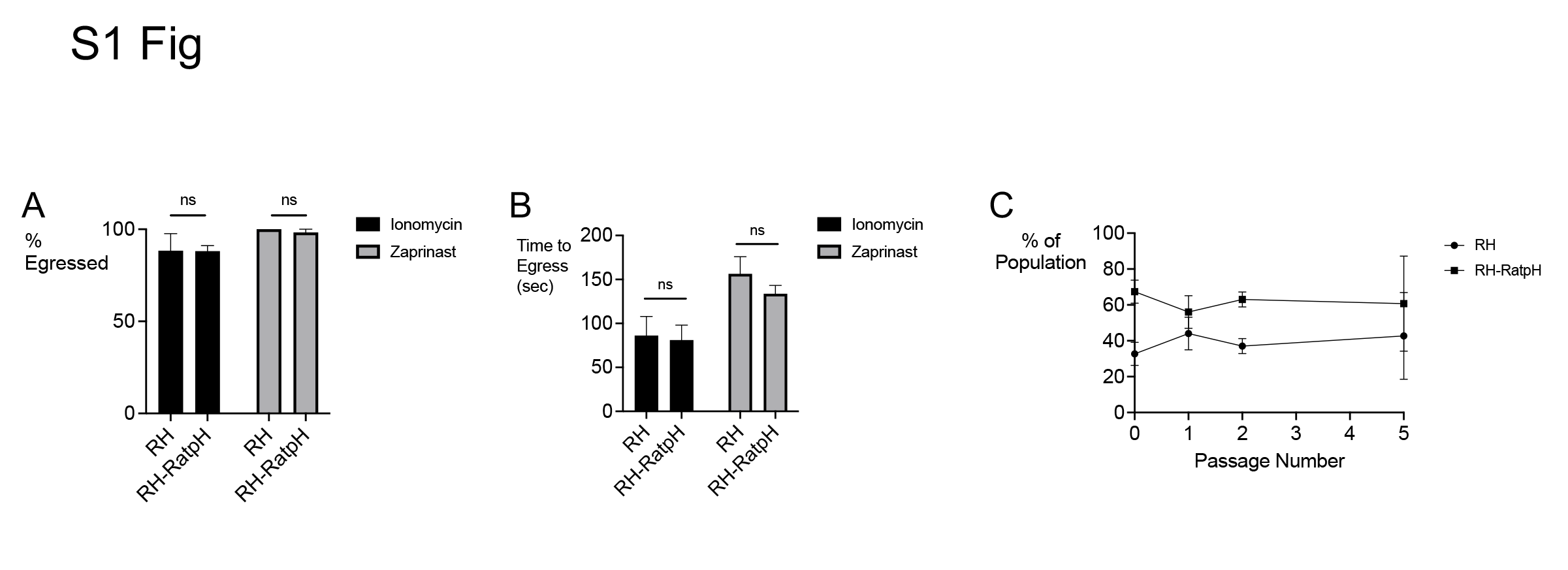

Supplement: S1 Fig — A) Analysis of egress efficiency after ionomycin or zaprinast induction. B) Measurement of time to egress after ionomycin or zaprinast induction. C) Analysis of parasite fitness, as measure by a co-culture competition assay wherein RH (37% of the starting population) and RH-RatpH (63% of the population) were enumerated after passages 1, 2, and 5 and expressed as a percentage of the total population. (TIF) [file ppat.1010139.s001.tif]

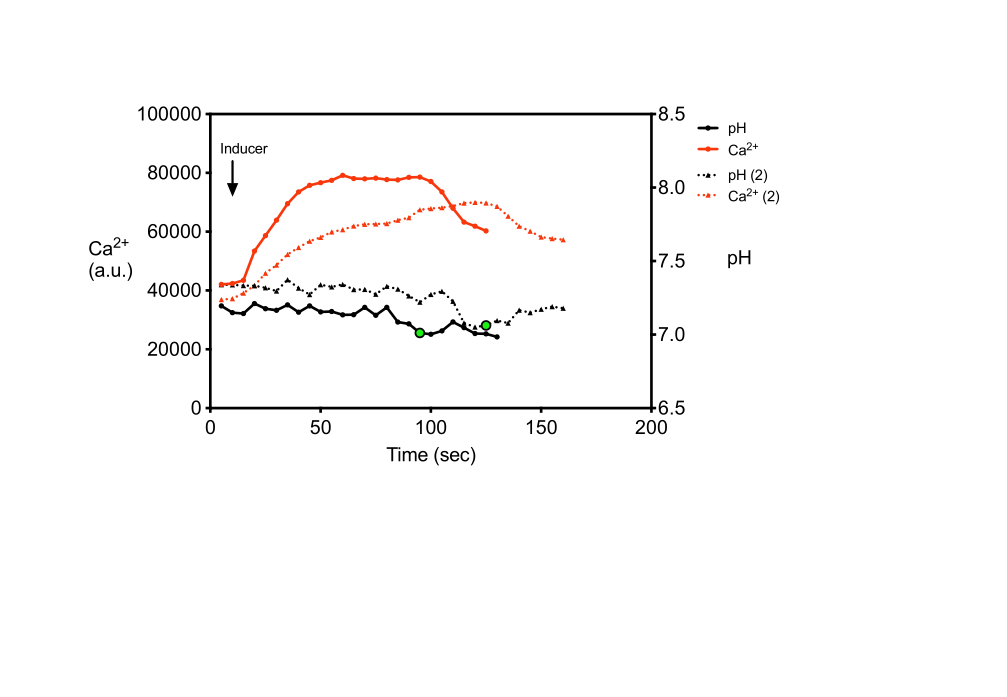

Supplement: S2 Fig — Green time points indicate PVM rupture. (TIFF) [file ppat.1010139.s002.tiff]

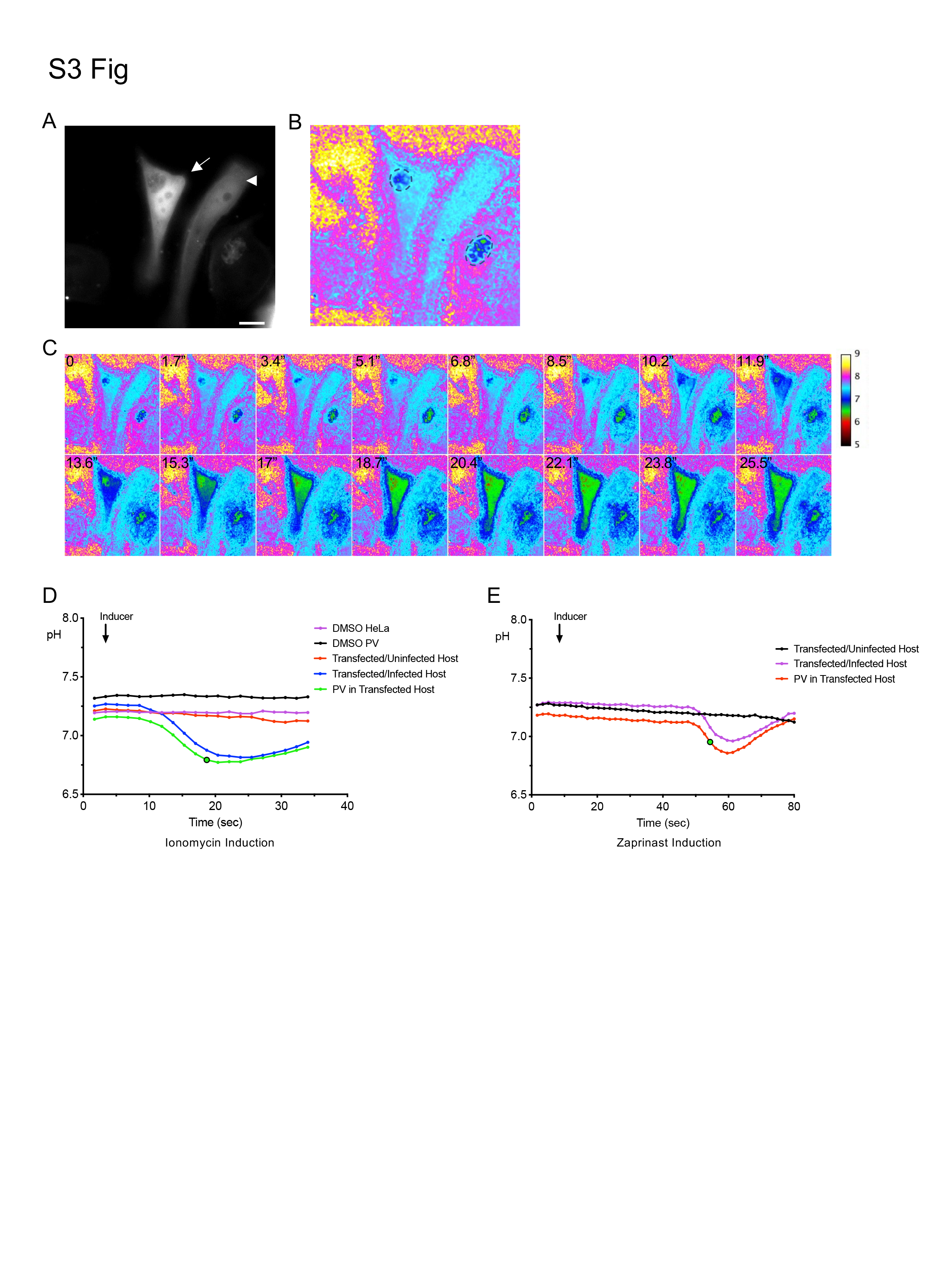

Supplement: S3 Fig — A) 410 nm image of HeLa cells transfected with RatpH. Arrowhead indicates a transfected HeLa cell and arrow indicates a transfected and infected (with RH-RatpH) HeLa cell. B) Ratio image (410/470 nm) of A), dashed lines indicate the vacuoles. C) Time-course of ratio images following ionomycin induction; time post-induction is indicated in the upper left corner of each image. D,E) pH tracings of infected and uninfected-transfected host cells or vacuoles induced with DMSO or ionomycin (D) or zaprinast (E). Scale bar, 10 μm. Green data points indicate PV rupture. (TIF) [file ppat.1010139.s003.tif]

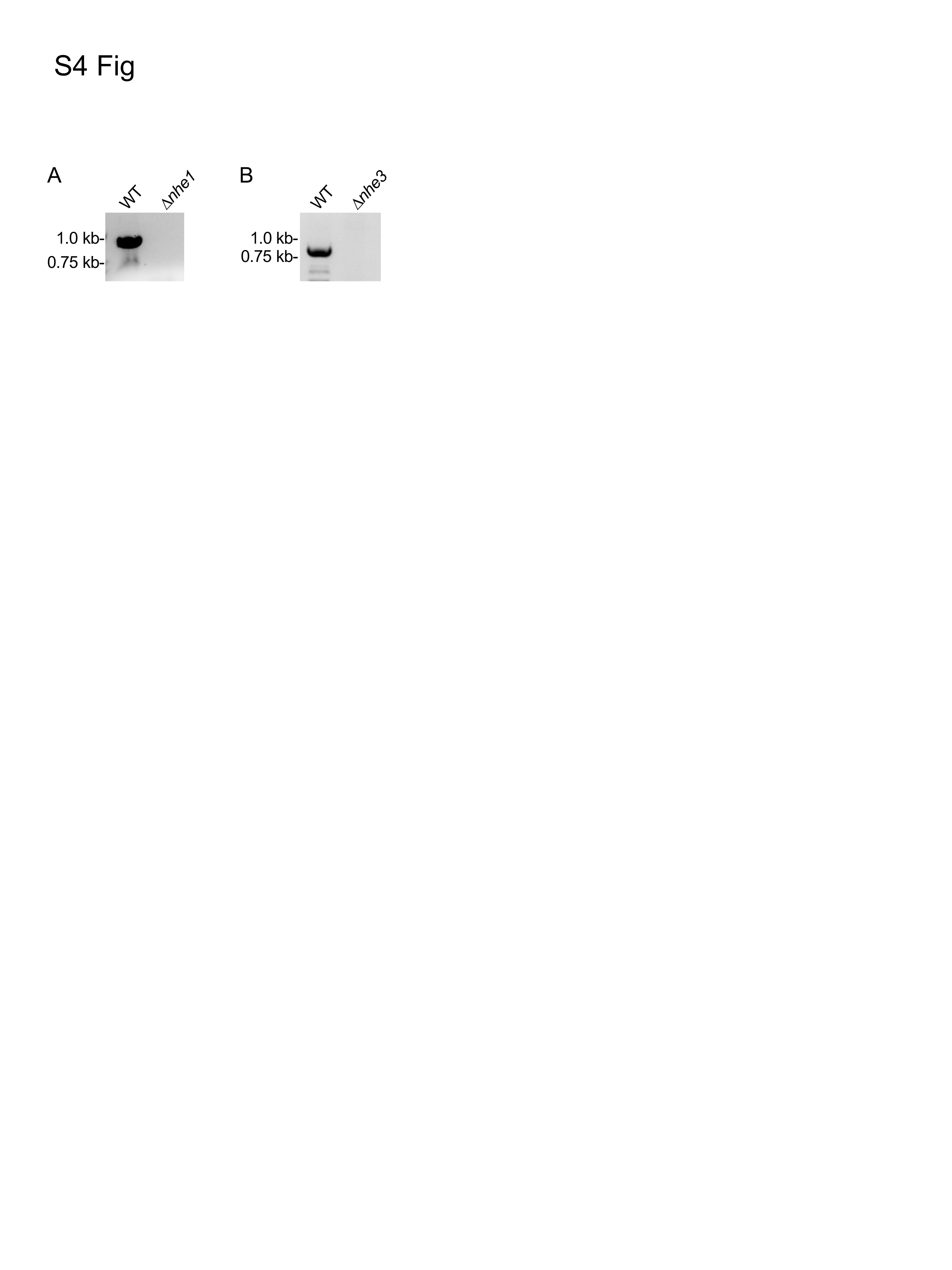

Supplement: S4 Fig — A) Primers designed to amplify a product only when a knockout of NHE1 has occurred (as used in (Arrizabalaga et al., 2004). B) Primers designed against the NHE3 gene detects NHE3 in WT but not Δnhe3 parasites. (TIF) [file ppat.1010139.s004.tif]

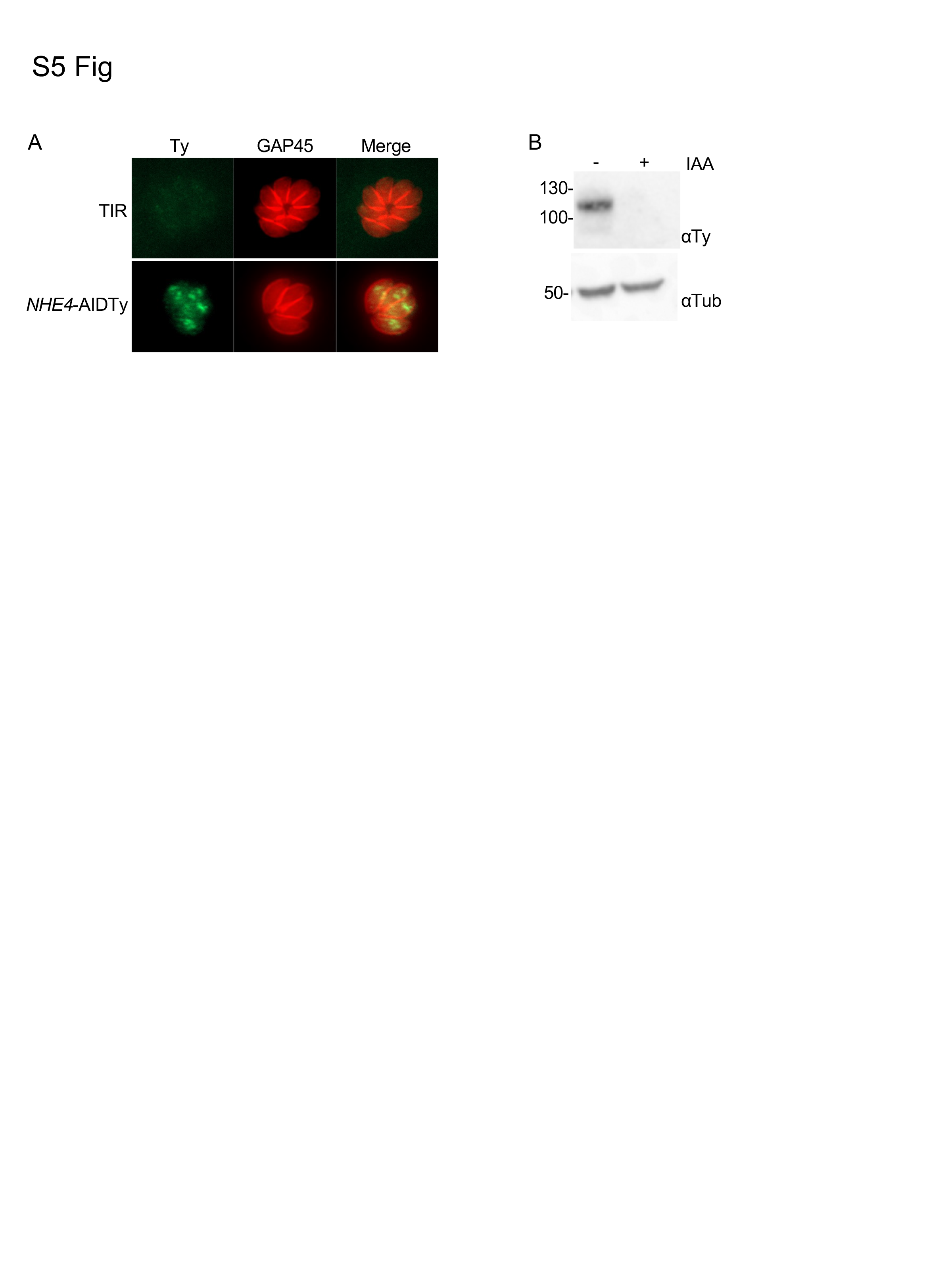

Supplement: S5 Fig — A) TIR or NHE4-AIDTy parasites grown in chamber slides for 24 h were fixed and stained with anti-Ty and anti-GAP45 antibodies. B) NHE4-AIDTy parasites treated for 24 h with or without IAA were purified and immunoblotted with anti-Ty. Immunoblotting with anti-tubulin was used as a loading control. Units represent apparent molecular weight in kiloDaltons. (TIF) [file ppat.1010139.s005.tif]

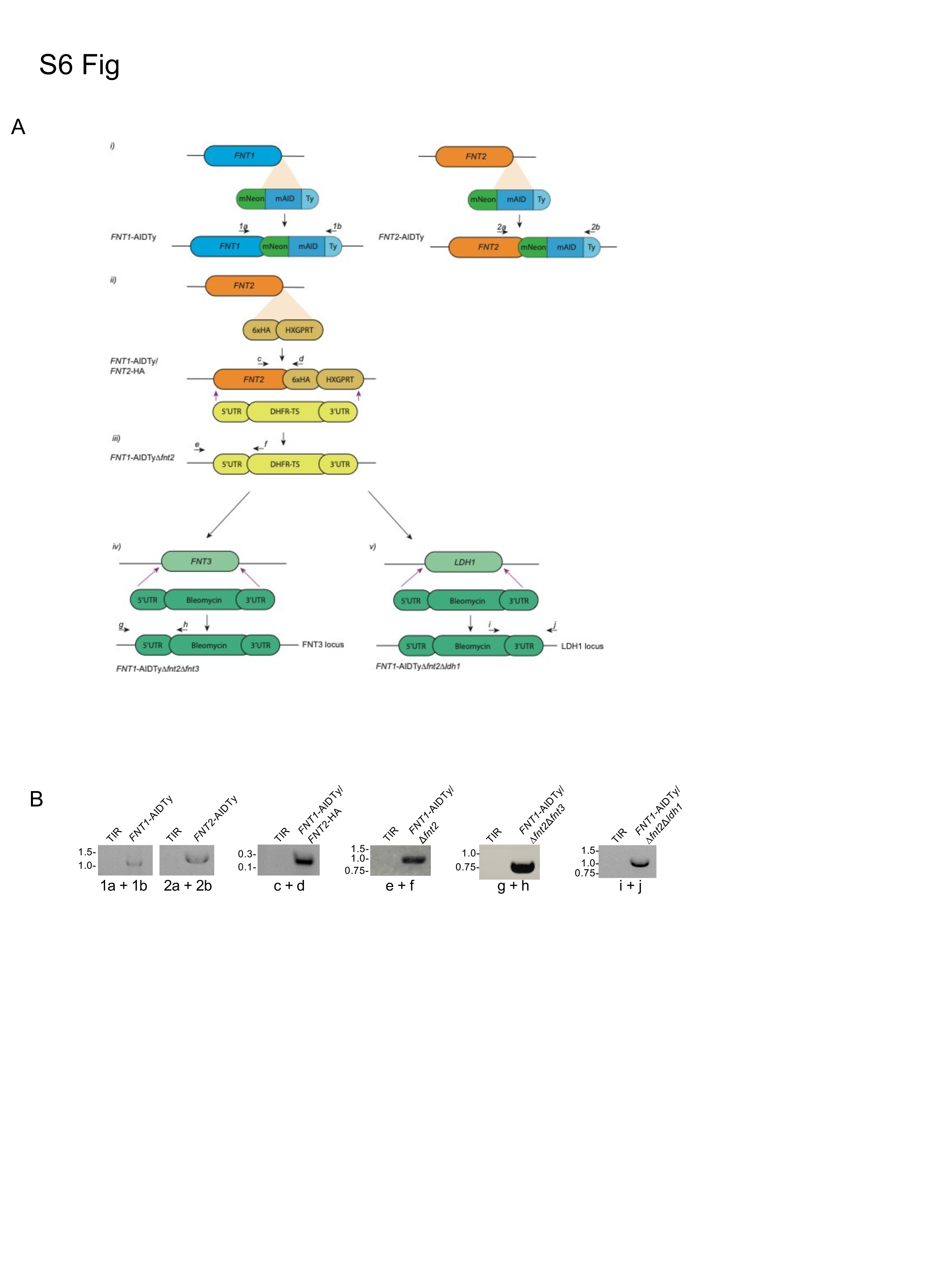

Supplement: S6 Fig — A) Schematic diagram of the strategies used to endogenously tag or knock out genes, and the lineage. B) PCRs to detect integration of epitope tags or selectable markers as indicated by the primers (shown as letters) in A. Units represent size in kilobase pairs. (TIF) [file ppat.1010139.s006.tif]

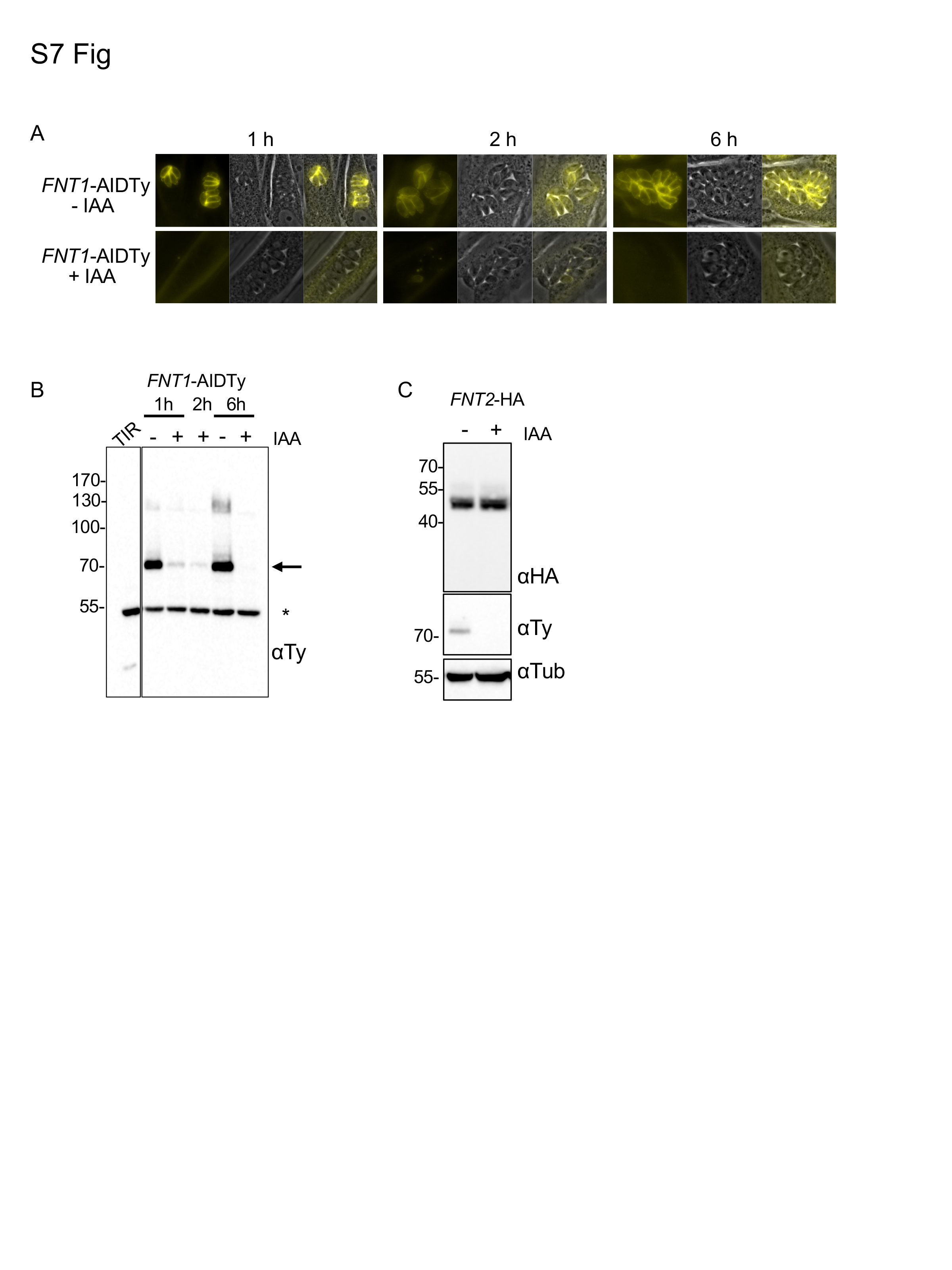

Supplement: S7 Fig — A) FNT1-AIDTy parasites were inoculated into Ibidi chamber slides and treated with or without IAA for the indicated times. The mNG fused to FNT1 was visualized with live imaging using a YFP filter cube. B) TIR and FNT1-AIDTy parasites were treated for the indicated times with or without IAA, filter purified, and lysates immunoblotted with anti-Ty. Arrow indicates FNT1-AIDTy and the asterisk indicates a non-specific band recognized by the Rb-Ty Ab. C) FNT2-HA tachyzoites were treated with and without IAA for 24 h and lysates immunoblotted for FNT2 (anti-HA), FNT1 (anti-Ty), and tubulin as a loading control. Units represent apparent molecular weight in kiloDaltons. (TIF) [file ppat.1010139.s007.tif]

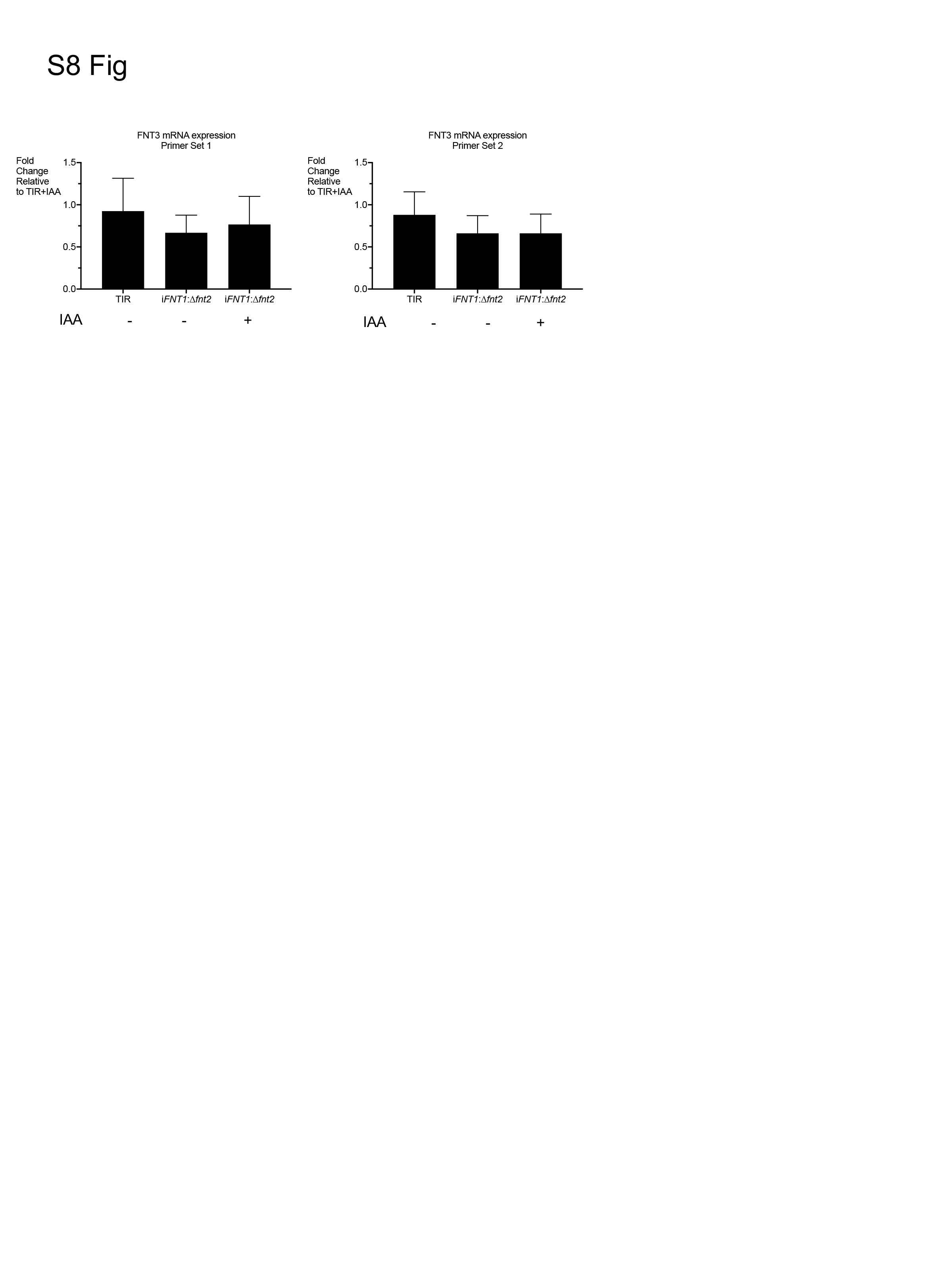

Supplement: S8 Fig — Two separate primer sets were used. Data represent 5 biological replicates each with triplicates samples. Error bars are mean ± S.E.M. (TIF) [file ppat.1010139.s008.tif]

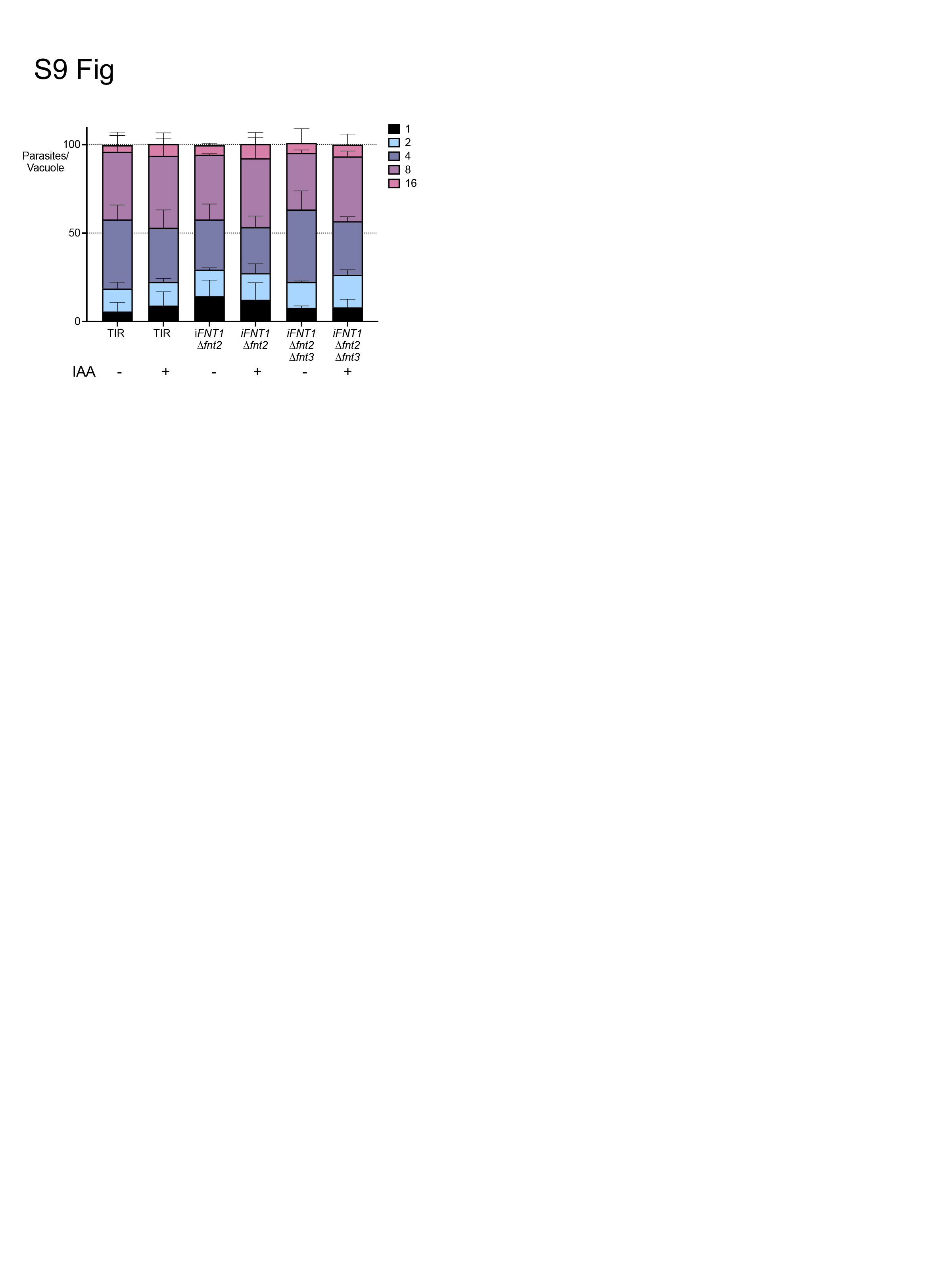

Supplement: S9 Fig — Parasites were inoculated into 8-well chamber slides and allowed to replicate for 24 h prior to enumeration. A minimum of 250 PVs were counted. Data represent 3 biological replicates each with triplicate samples. Error bars are mean ± S.E.M. (TIF) [file ppat.1010139.s009.tif]

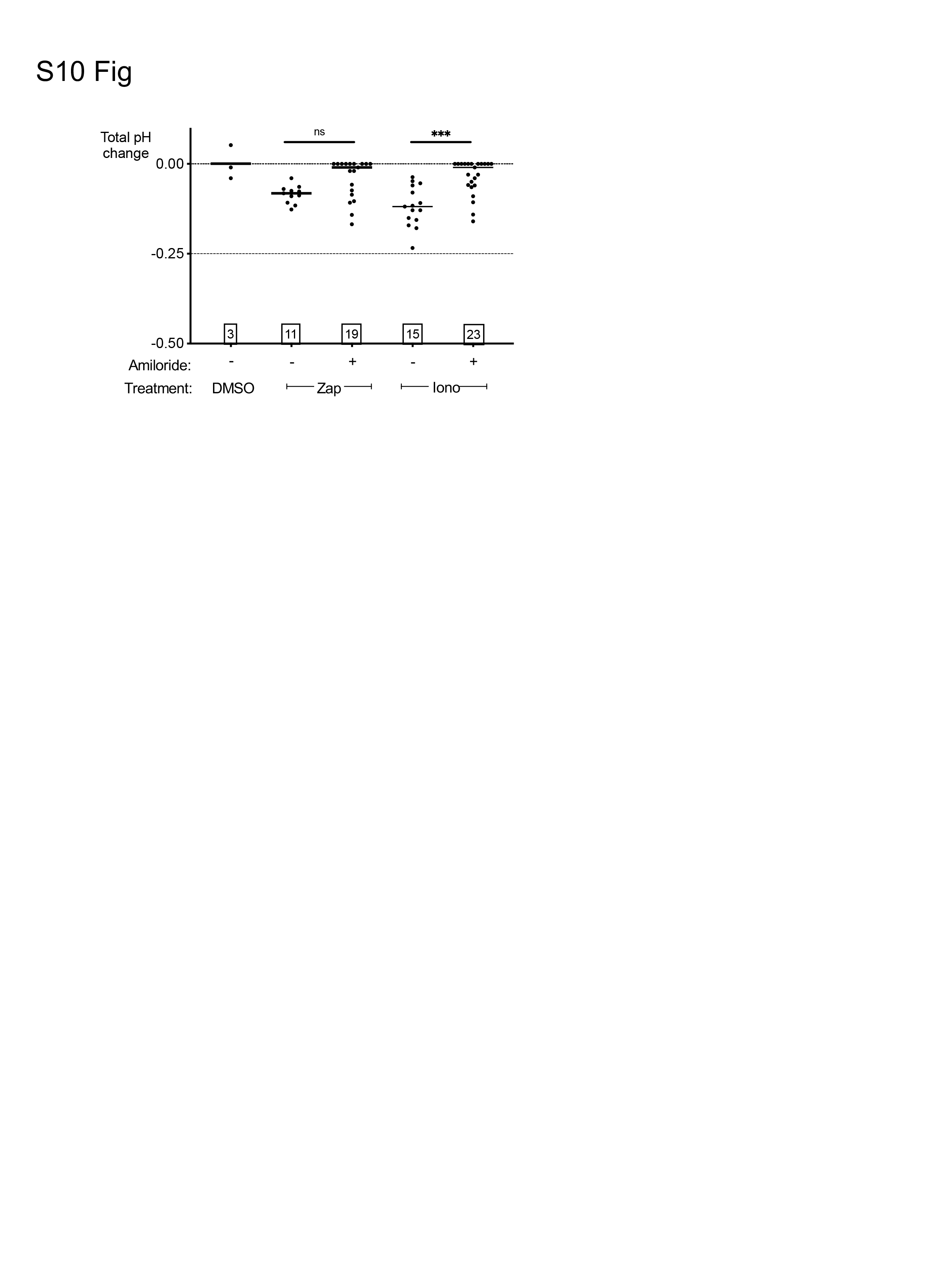

Supplement: S10 Fig — RH-RatpH vacuoles incubated with or without amiloride. Data points represent changes in PV pH starting from baseline to a drop greater than 0.05 following induction with either ionomycin or zaprinast. (TIF) [file ppat.1010139.s010.tif]
