# Supplementary material for: Toxoplasma gondii excretion of glycolytic products is associated with acidification of the parasitophorous vacuole during parasite egress
Source: PLoS Pathog. 2022 May 5;18(5):e1010139. doi: 10.1371/journal.ppat.1010139 (PMC9113570; doi:10.1371/journal.ppat.1010139)
Supplement: Attachment — Submitted filename: Response to reveiwers_20220307.pdf [file ppat.1010139.s016.pdf]

We thank all the reviewers for their thoughtful comments and suggestions, which are addressed in the following point-by-point responses.

Part I - Summary

Please use this section to discuss strengths/weaknesses of study, novelty/significance, general execution, and scholarship.

Reviewer #1: This historically narrated study reports elegant new reporters and techniques, with many new mutant parasites lines and experiments performed, however, only the last figure displays a modest set of data indirectly correlating the release of glycolysis products with vacuole acidification. Despite the large amount of work, progressive insight into the underlying mechanism is not well developed; although the presented data toward such a connection is intriguing, it presents many more loose observation points than lines that can connect them. Furthermore, data presentation and application of statistics are not consistent and not always correctly interpreted (notably, the details regarding measurement and interpretation of CytD/MycD use is not well presented). With that said, the imaging is very well done and tools are beautiful, which makes this more a tool development report than a major biological advance, or at least, premature.

We thank the reviewer for identifying strengths and limitations of the work. Unfortunately, directly tying the release of glycolysis products to PV acidification is not possible due to the lack of specific imageable biosensors for such products. Accordingly, the conclusions with respect to glycolytic products contributing to the acidification of the PV during egress are based the observations that both PV acidification and the release of glycolysis products are attenuated upon genetic or chemical disruption of FNT1 and/or FNT2. Also, our finding that a variety of other proton transporters do not measurably contribute to PV acidification centers the focus on FNTs and their export of glycolytic products. Additionally, glycolysis is known to be the main source of ATP for parasite motility, which is activated during egress. Together these findings support a model in which glycolysis is activated to drive egress and to moderately acidify the PV via the export of glycolysis products. We recognize that our findings do not provide a complete picture of how the PV acidifies during egress. However, we emphasize that the study provides novel insight into the sequence of events that occur during egress. Also, the revised manuscript includes new findings that expose for the first time a role for PLP1 in Ca^{2+} influx from the medium to the host cytosol during a step that precedes its previously described role in PV rupture. The latter observations provide an important missing link in the sequence of events, one that likely explains why parasites lacking PLP1 show aberrant Ca^{2+} signaling during egress. Finally, we have addressed the inconsistencies in the presentation, statistical analysis, and interpretation of data including that for CytD and MycB. We appreciate the reviewer’s time in reevaluating the revised manuscript along with considering the other reviews and our responses to them.

Reviewer #2: In this study from Huynh and Carruthers, the authors attempt to address the contribution of acidification to parasite egress. Following up on their earlier work (Roiko et al, 2014), they use an improved pH-sensitive genetic reporter for better quantitation and interrogation of the event. They definitively confirm that PV acidification precedes both natural and stimulated egress, and that acidification is PLP1 dependent. These data are interesting, and strongly supported by their data. The authors then seek to identify the molecular basis and origin of acidification, providing a detailed analysis of ionic contribution, as well as proton pump candidates with an extensive genetic interrogation of a panel of potential molecular contributors.

Recommendation:

The experiments are carefully done (though the supp. figures need polishing), but some major concerns remain into the strength of their conclusions on the basis of the data they present, and the logic in the current narrative. These are detailed below. While these are major concerns, the contributions of the manuscript are sufficiently important and interesting that a revised manuscript should be considered for publication.

Reviewer #3: In this study, the authors investigated the acidification of the parasitophorous vacuole during egress of Toxoplasma gondii tachyzoites. For this purpose, they developed a novel assay based on genetically encoded ratiometric pHluorin (RatpH), which allows to monitor absolute variations in pH in live imaging experiments. Using these transgenic parasites, the authors document a drop of PV pH occurring immediately prior to induced and natural egress. When induced egress experiments were performed with plp1ko parasites expressing RatpH, an attenuation of the PV pH drop was observed, and a lack of acidification was even observed when motility was blocked (by cytD) in the plp1 mutant. The authors then tested several parasite transporters that could participate in acidification of the PV, using genetic approaches. They found that formate-nitrite transporters FNT1 and FNT2 contribute to acidification of the PV during egress, and that other unidentified factors also participate. Finally, based on analysis of extracellular tachyzoites, the authors conclude that release of lactate and pyruvate contribute to acidification of the PV during parasite egress.

Overall, the paper is very well written and clearly presented. The core of the study relies on a robust and elegant approach to measure pH using genetically encoded ratiometric pHluorin, allowing live imaging of PV pH during parasite induced or natural egress. The data provide definitive evidence that pH drops prior to egress. The study also includes a thorough analysis of genes potentially contributing to PV acidification, with single, double and triple mutants, using both conventional and conditional genetic strategies.

While a previous study from the same group showed that low pH promotes PLP1 secretion and activity (Roiko et al 2014), the authors now propose that PLP1 contributes to acidification of the PV, although the underlying mechanism remains unclear. However, this part of the work lacks important controls, as detailed below, to strengthen the conclusion that plp1 depletion attenuates the drop in PV pH. Furthermore, the relevance of using ESA from extracellular tachyzoites to measure lactate and pyruvate release is not obvious.

Part II – Major Issues: Key Experiments Required for Acceptance
Please use this section to detail the key new experiments or modifications of existing experiments that should be <u>absolutely</u> required to validate study conclusions.

Generally, there should be no more than 3 such required experiments or major modifications for a "Major Revision" recommendation. If more than 3 experiments are necessary to validate the study conclusions, then you are encouraged to recommend "Reject".

<u>Reviewer #1</u>: (No Response)

<u>Reviewer #2</u>: Major comments:

1. These genetically encoded reporters can buffer the event they are intended to report upon, and consequently affect cell physiology. This should be addressed by plaque assay for the reporter lines to show no gross different in parasite health as a consequence of expressing these reporters. Alternatively, a competition growth assay could be used to address this point.

These are important considerations. To determine if expression of pHluorin affects parasite physiology, we compared RH and RH-RatpH parasites for egress efficiency and the timing of egress following ionophore induction. We also measure parasite replication via co-culture in vitro competition assay. The results, which are presented in a revise S1 Fig, indicate no differences between RH and RH-RatpH in parasite egress or replication.

2. Line 248: 'RH-RatpH-RGECO parasites displayed a rapid increase in cytosolic Ca2+ after ionomycin induction and well before the drop in PV pH and egress (Figure 3F and Supplemental Figure S2).' While I agree with the author's interpretation of the data, the major challenge of comparing signal outputs from different reporters is that the kinetics of signal detection and integration of the signal input for the reporter output are likely to be different. Which makes comparison of the two reporter signals inherently unfair. This should be more cautiously worded throughout, and this caveat noted in the discussion. Also, the speed at which the camera acquires the images (and channel order in which they are acquired) could

affect the interpretation of the data, and possible result in an experimental artifact. To address this the authors should switch the order of channel acquisition to confirm that there is a genuine difference in the timing of the events.

We repeated the measurement of the RGECO and pHluorin signals by acquiring the RGECO signal first followed by the pHluorin ratiometric signal, and then switching the order to measure pHluorin followed RGECO. The results, which are shown in a revised S2B Fig, indicate that the timing of Ca^{2+} increase and PV pH decrease relative to one another are the same regardless of the order of signal acquisition. In other new experiments, we measured host Ca^{2+} with two different indicators (RGECO and Cal-590) and observed similar results. We also simultaneously measure host Ca^{2+} and parasite Ca^{2+} with the same indicator (RGEGO), thereby avoiding a potential pitfall of differential response rates due to using different indicators.

3. The experimental logic and rationale needs elaboration, as at the present time some sections are somewhat contradictory. My main point of confusion comes from the following: lines 225-229: 'However, PVs of immobilized Δplp1-RatpH showed no decrease in pH and they failed to release pHluorin for the entire duration of the experiment (up to 20 min) in all 225 vacuoles observed (Figure 3E). Taken together, these findings suggest that PLP1 influences PV pH and that rupture of the PVM and possibly the host plasma membrane via PLP1 or motility is necessary for acidification of the PV'. My reading of this that microneme release of PLP1 is necessary for the process of acidification. The primary function of PLP1 is thought to be the formation of pores on the parasitophorous vacuole membrane, ultimately leading to parasite egress. This would indicate that acidification is likely host-cell dependent, taking place on the PVM. Following the PLP1 dataset (Fig. 3), the authors explore the molecular basis of acidification: Line 257: 'Several plasma membrane proton transporters are not required for acidification of the PV' – if acidification is PLP1-dependent (which they convincingly demonstrate), and PLP1 is thought to be PVM localized following its release from the micronemes, why do the authors then explore parasite plasma membrane transporters? It is possible that this is simply an issue with the manuscript narrative structure – but as it's currently written it is difficult to follow the logic of their work, and understand why this was done.

The reviewer raises an important point that we agree needed attention. In essence the issue is that PLP1 is known to rupture the PV; however, PLP1 is also required for normal Ca^{2+} signaling in the parasite (Vella et al 2021) and for normal PV acidification (our work), which both occur prior to PV rupture. What we were trying to articulate (rather vaguely, we admit) is that there must be a role for PLP1 upstream of the potentiation of parasite Ca^{2+} and PV acidification. Because parasite Ca^{2+} potentiation depends on host Ca^{2+} that transiently increases during egress (Vella et al 2021), we reasoned that PLP1 could play a role in initiating the transient increase in host cytosolic Ca^{2+}. We further posited that because extracellular Ca^{2+} is necessary for normal acidification of the PV (our work), this could indicate a role for PLP1 in Ca^{2+} entry into infected host cells from the media. To test this, we infected RGECO host cells with RH-RatpH-RGECO parasites, thereby allowing us to simultaneously monitor host Ca^{2+}, parasite Ca^{2+}, and PV pH. In medium containing Ca^{2+}, we observed the expected initial elevation of parasite Ca^{2+} due to zaprinast induction that was later followed by simultaneous elevation of host Ca^{2+} and potentiation of parasite Ca^{2+}. We also found that PV acidification is concurrent with the elevation of host Ca^{2+} and potentiation of parasite Ca^{2+}. Similar results were seen in cells infected with RH-RatpH and loaded with a synthetic Ca^{2+} indicator (Cal 590-AM). However, in medium lacking Ca^{2+}, we observed markedly tempered elevation of host Ca^{2+}, limited potentiation of parasite Ca^{2+}, and attenuated acidification of the PV. Collectively, these findings suggest that extracellular Ca^{2+} is necessary for host and parasite Ca^{2+} signaling along with PV acidification in conjunction with parasite egress.

To determine if PLP1 and/or motility are required for host Ca^{2+} signaling, we measured host Ca^{2+} in cells infected with RH-RatpH -/+ cytochalasin D (CytD) treatment and with Δplp1-RatpH -/+ CytD treatment. In cells infected with RH-RatpH parasites (without CytD), we again found that host cytosolic Ca^{2+} increased prior to PV rupture. A similar increase in host cytosolic Ca^{2+} was seen for CytD immobilized RH-ratpH parasites, indicating parasite motility is not required for host Ca^{2+} signaling when PLP1 is present. Cells infected with motility competent Δplp1-RatpH parasites showed Ca^{2+} signaling prior to PV rupture, albeit with a considerable delay during which parasite motility was observed, indicating that parasite motility can trigger host Ca^{2+} signaling in the absence of PLP1. Finally, and crucially, immobilized parasites lacking PLP1 showed no host Ca^{2+} signaling, indicating that in the absence of motility host Ca^{2+} signaling critically depends on expression of PLP1. Taken together our findings confirm a requirement for PLP1 for normal Ca^{2+} signaling in the parasite, which is depended on host Ca^{2+} signaling resulting from

influx of extracellular Ca^{2+}, all of which occurs before PV rupture. These findings indicate a critical role for PLP1 (or parasite motility) in initiating host Ca^{2+} signaling and the ensuing cascade of events leading to parasite egress. We appreciate the reviewer bringing attention to this apparent conundrum since addressing it has exposed a missing link between PLP1 and initiation of egress, one that is now incorporated into the working model in Fig 8.

4. Line 304: 'We initially tested the concentrations of the inhibitors that were used in the previous study, but 10 μM, BH-296 blocked tachyzoite egress, precluding its use at this concentration' – the basis for this is unclear. The authors previously use CytD and MycB to 'paralyse' parasite and remove variation that can be introduced into the microscopy studies by motile parasites. Treatment of these parasites with these paralyzing drugs prevents egress but does not prevent the authors from assessing changes in pH. This discrepancy needs to be addressed.

This is another excellent point for which we were unclear in our explanation. Because CytD and MycB block motility and prevent parasite egress, it was necessary to use PV rupture (indicated by a decrease in pHluorin signal) as a proxy for "egress". To be consistent we used PV rupture as the temporal reference point for all the live imaging experiments. We now describe this more clearly in the text. Regarding BH-296, prior to testing it (and BH-388) in live imaging experiments, we had initially used a static egress assay with overnight inhibitor pretreatment of infected cells. Parasites treated with 10 uM BH-296 were severely affected in their growth (presumably due to lactic acid toxicity; Kloehn et al., 2020) and did not respond to ionophore-induced egress. We subsequently reduced the incubation time with the inhibitors to 2 h for the live imaging data that was included in the first submission, but we did not test 10 uM BH-296 in those experiments. The reviewer's comment prompted us to revisit testing 10 uM BH-296. The results, which are now included in Fig 6A, indicate that 10 uM BH-296 treatment substantially attenuates acidification of the PV, thereby adding to the evidence that FNT-dependent export of glycolysis products contributes to PV acidification preceding egress.

5. Line 416: 'Parasites lacking all three FNTs (iFNT1Δfnt2Δfnt3+IAA) still showed residual release of pyruvate, implying the existence of other unidentified transporters capable of exporting pyruvate.' As FNT1 is a conditional knockdown and not a clean knockout, the formal possibility remains that residual FNT1 is responsible – and that there is no need to imply the existence of other pyruvate transporters. This should be addressed in the text.

This has been added as a possible explanation for the residual pyruvate release, lines 1039-1041.

6. Line 453-455: 'Nevertheless, a drop in pH is not essential for exit from host cells, which is consistent with the event being regulated at multiple levels to ensure successful liberation.' Do you mean is essential? Otherwise, I'm not sure the logic of the sentence is consistent, or is confusing as currently written – it is not clear if 'the event' refers to the drop in pH or egress.

The event refers to egress, which has been clarified in the text, line 1089.

<u>Reviewer #3: Major comments</u>:

1. The plp1 mutant experiments lack an important control in figure 3B/C, the non-induced condition (DMSO). How could the authors determine the difference in lowest regional PV pH for the plp1 mutant without having the non-induced condition (Line 216-217)? In fig 3F, the pH in plp1 mutant seems to be higher than RH. Could this explain the apparent lower pH drop in fig3B/C, where comparison was made with the non-induced RH parasite? In Fig3E, pH drops constantly in the plp1ko with cytD/MucB conditions, even before the addition of the inducer (ionomycin), suggesting a potential experimental issue.

We had performed the Δ*plp1* DMSO controls previously but did not include data from them in the graph because there is essentially no change in the PV pH after DMSO treatment. In hindsight we recognize that some of the misunderstanding might be due to insufficient explanation of how we calculate pH changes from the tracings. To address this, we have added the following sentence to the results section:

"To quantify the decrease in PV pH from the tracings, we took the lowest pH value, which typically occurred near the time of PV rupture (identified by pHluorin release) and subtracted it from the

pH value that was the highest immediately prior to the descent, as described more extensively in the Materials and Methods."

We have also added the following description to the Materials and Methods:

"To quantify the decrease in PV pH from the tracings, we identified the lowest pH value and subtracted it from pH value that was the highest immediately prior to the descent. If no obvious descent was observed (i.e., as seen with some treatments and mutants described below) then we took the pH value from the time of PV rupture and subtracted it from the value measured 15 sec earlier. Since DMSO treated samples showed no clear descent and they lacked PV rupture as a reference point, we calculated the average time to PV rupture for ionomycin induction and used it as the reference point for measuring PV pH relative to the value measured 15 sec earlier."

Additionally, we went back and calculated the values for Δ*plp1* DMSO, both with respect to change in PV pH and lowest PV pH. This data is now included in Figs 3B and 3C. The data also establish that there is no difference between DMSO treated RH-RatpH and Δ*plp1*-RatpH parasites with respect to change in PV pH or lowest PV pH.

Drift in the PV pH tracings was not observed consistently and was not evident in most of the PVs that were measured in the initial set of experiments. To confirm this, we performed additional experiments and new examples are shown in Figs 3D, 3E, 3F, and 3G.

2. The relevance of using ESA to analyze lactate/pyruvate transport is questionable (figure 6). ESA fractions likely represents components released upon induced exocytosis of apical organelles. FNTs or other transporters are expected to act at the parasite plasma membrane, therefore the relevance of the ESA experiments is not clear. This probably explains why the results are inconsistent with the Zeng et al study, which analyzed transport across the parasite plasma membrane. Because of this limitation, it is difficult to conclude on the contribution of lactate/pyruvate in acidification of the PV. The experiments using ldh1 mutants suggest that lactate production is not required for acidification of the PV. How then could lactate release contribute to PV acidification, as proposed by the authors?

Although the analysis of supernatant (ESA) from extracellular parasites is not a direct measurement of the products released during parasite egress, it acts as a surrogate since there is no method or assay to measure the contents of the PV prior to or following egress. Also, Kloehn et al., 2020 analyzed supernatant from extracellular parasites lacking FNT-1, BCKDH, or FNT-1 and BCKDH to measure parasite release of metabolites including lactate, thus setting a precedent for such an analysis. We have added this reasoning in lines 991-994.

Zeng et al. measured lactate transport across the parasite plasma membrane, but they did so in the opposite direction of what would occur naturally. We do not bring attention to this in our manuscript, but it is possible that FNTs have differential transport properties in each direction, thereby also providing a potential explanation for the apparent discrepancy.

Our experiments show that pyruvate is still released by the ldh1 mutant, thereby providing a plausible explanation for why this mutant isn't entirely defective in PV acidification. Also, because we cannot disrupt excretion of lactate and pyruvate entirely, the extent to which PV acidification is due to residual release of these products (with co-transport of H^+) or release of H^+ via other means remains to be determined.

Finally, we now recognize that because "Excreted/Secreted Antigens (ESA)" specifically refers to antigens, this term is not appropriate when referring to metabolites. Accordingly, we use the more agnostic term "supernatant" instead of ESA in the revised manuscript.

Part III – Minor Issues: Editorial and Data Presentation Modifications

Please use this section for editorial suggestions as well as relatively minor modifications of existing data that would enhance clarity.

Reviewer #1: Minor comments:

1. Line 57 "PV pH during egress" should this be 'chemically induced' or 'pharmacologically triggered' egress, since a DMSO control is mentioned in the second half of the sentence, but it is not clear what this controls for.

"ionophore or zaprinast-induced" has been added to line 58.

2. Line 64 “lead an abatement” should this be ‘lead TO an abatement”?

“to” has been added to line 64.

3. Legend Fig 1D. please add that ionomycin was used as egress inducer

“ionomycin” has been added to line 1631.

4. Fig 2; 3C. The horizontal line represents the mean, however, the Kruskal-Wallis + Dunn measures the ‘median’. This seems therefore to be a mismatch in graphical presentation and statistical analysis.

All graphs using the Kruskal-Wallis test now indicate “median”.

5. Fig 2B. The schematic says “zaprinast’ this should be ‘ionomycin’

This has been corrected in the figures.

6. Line 190: “Since CytD did not markedly affect PV pH…” the statistics for that is not shown, and there appears to be a shift to more aciditiy upon additions of CytD. Neither is a control of just CytD included.

The statistics for comparing with and without CytD for both zaprinast and ionomycin induction are now shown on the graph.

7. Fig 3B. It is not indicated whether the line represent mean or median

It is now indicated that the bar represents the median, line 1708.

8. Fig 3C. Assumingly, the Kruskal-Wallis + Dunn analysis was also applied here.

It is now indicated that the Kruskal-Wallis test was performed and bar represents the median, line 1708.

9. Fig 3D, E. Both CytD and MycD display a gradually decreasing pH of about 0.5 units that equilibrates around 300 sec in (Fig 3E). As such, when the measurement was performed in Fig 2 in presence of CytD would have a major impact on experimental outcome (see #6 above as well). It was said 5 min in the M&M. Assuming the traces shown in Fig 3D, E are representative, it is surprising to see in Fig 2A, B that the average/median (?) pH drop in Fig 2A/B is less than 0.25. So somehow these observations do not add up to exactly the same thing in these two experiments.

We apologize for the confusion stemming from the drift of signal seen in some examples where no clear changes in PV pH occurs and for the lack of clarity of how we calculated a change in PV pH. To address this, we repeated experiments for several of the conditions including for DMSO controls, and we provide in the revised manuscript more representative traces in Figs 3D and E. Also, in the Materials and Methods we explained more thoroughly how changes in pH were measured from the tracings:

“To quantify the decrease in PV pH from the tracings we identified the lowest pH value and subtracted it from pH value that was the highest immediately prior to the descent. If no obvious descent was observed (i.e., as seen with some treatments and mutants described below) then we took the pH value from the time of PV rupture and subtracted it from the value measured 15 sec earlier. Since DMSO treated samples showed no clear descent and they lacked PV rupture as a reference point, we calculated the average time to PV rupture for ionomycin induction and used it as the reference point for measuring PV pH relative to the value measured 15 sec earlier.” lines 1333-1340.

10. Fig 4C-F. It would be helpful to also mark which comparisons are ‘not significant’, as (partly) done in 2B, 3C, 5A,E,F,H

“not significant” (ns) has been added to these graphs.

11. Line 306 states “At both 1 uM and 5 uM, BH-296 reduced the magnitude of pH change during zaprinast-induced egress compared to RH-RatpH, …” However, in Fig 5A only 5 uM BH-296 is statistically significant, not 1 uM.

These experiments were repeated with 10 uM BH-296 and the new data are now presented in Fig 5A. It now states that only 10 uM of BH-296 is statistically significant, line 816.

12. Text makes not reference to Fig 5E before mentioning Fig 5F

This has been added to line 924.

Reviewer #2: Minor comments:

1. Figure 1E – it would be useful to include an indicator on the trace for the point of egress to be able to relate trace shifts with the physiological event.

The data points that are colored green indicate the point of egress, which has been added to the graph and made clearer in the legend, line 1621.

2. Line 232-233: 'ratiometric pHluorin under a mammalian promoter' – should be reworded for clarity – 'under a mammalian promoter' is meaningless.

We realized that it is not necessary to indicate the type of promoter at this point in the text and instead have provided this information in the Materials and Methods, lines 1241-1242.

3. Fig. 3D and E: from the traces presented it appears that the starting pH of RH and Δplp1 vacuoles are different – is this correct? Were vacuoles containing equal numbers of parasites compared? This should be discussed.

These experiments were repeated, and better representative tracings are now shown in Figs 3D and E, showing similar starting pH of RH and Δplp1 vacuoles. We have also included a statement in the Materials and Methods that vacuoles containing at least 8 parasites were enumerated for all experiments, line 1301.

4. The presentation of data in 5D and E is confusing – it would be better to indicate the DMSO lane are also RH parasites – this could be done with a second +/- line indicating the use of an egress inducer in that experimental condition, and relabeling DMSO to be RH.

The graphs have been reformatted to include a line for inducer.

5. Line 251-254: regarding Fig. 3G – please provide details of statistical test used.

Student's t-test has been added to line 1700.

6. Line 265: 'generated in Stasic et al' – the correct numerical reference has been missed.

This reference has been added.

7. Figure 5C: the panel strain label has been incorrectly cropped off.

The figure has been re-cropped.

8. Figure 7: 'BCKDH, branched chain ketoacid dehydrogenase' is included on the figure and in the legend, but not mentioned in the manuscript – please remove for simplicity or explain its inclusion.

Upon revising this figure, which is now Fig 8, we realized that it is unnecessary to depict BCKDH in the figure. We have removed it accordingly.

9. Figure S1: add scale bars

This has been added to the figure.

10. Figure S2: add scale bars. also, for the pHluorin movie, the pH colored scale bar is misleading as the colors associated with each pH seem to change between time points. This means that a given color does not consistently represent a specific pH, and when viewing the data as a time series movie it makes it almost impossible to interpret the data real time. This should be corrected to have the colors used be consistent across the image series.

This appears to be an artifact generated during the conversion to a gif since the avi file and the individual tiff images do not shift in the scale bar. A static scale bar has been added to the gif to avoid confusion.

11. Figure S3: add units for the numbers presented on the gels.

This has been added to the gels.

12. Figure S4: add scale bars. Add units for the numbers presented on the gels.
Units for the western blot has been added to the figure legend, line 1925.

13. Figure S5b: add units for the numbers presented on the gels.
Units for the DNA gels has been added to the figure legend, line 1929.

14. Figure S6: add scale bars. Add units for the numbers presented on the gels.
Units for the western blot has been added to the figure legend, line 1938.

Reviewer #3: Minor comments:

1. The number of PV analyzed to generate “representative tracings” should be specified in the figure legends (fig 3 and fig 4).
We have made it clearer that the representative tracings are examples from the graphs associated with those figures, line 930, 956.

2. The data show that FNT2 cannot be detected in iFNT2 or FNT2-HA parasites. Is there a compensatory overexpression of FNT2 in FNT1-depleted parasites? Could this explain why the combined depletion of both FNTs is effective in contrary to single knockdowns.
Western blot using anti-HA to detect FNT2 in FNT1kd parasites (FNT1-AIDTy/FNT2-HA) -/+ IAA.
We addressed this concern by looking at the expression of FNT2-HA in FNT-depleted parasites and saw no upregulation of FNT2 expression. This is shown in S7C Fig and in the text, lines 927-929.

3. Line 205-207: it I unclear how PLP1 and PVM breakage could contribute to ion flux to acidify the PV. Is it from the host cell? More generally, the model in fig7 would benefit from the addition of a temporal dimension specifying the sequence of events, as nicely done in the discussion.
As described more extensively in response to reviewer 2 (see major comment 2 above), we performed additional experiments that revealed a role for PLP1 or motility in host Ca^{2+} signaling resulting from influx of Ca^{+2} from the extracellular medium. Also, using the Ca^{2+} indicator Cal-590 or host cells expressing the genetically encoded RGECO Ca^{2+} indicator, we show that host cytosolic Ca^{2+} consistently increased prior to egress/pHluorin release. The new data is now presented in Fig 4. Taken together with findings from other recent work (Vella et al.,2021), our findings are consistent with PLP1 (or motility) playing an early role in egress by dictating the influx of Ca^{2+} from the medium into host cells as an initial event that is connected to parasite Ca^{2+} signaling and PV acidification, all of which occurs prior to PV rupture. Precisely how PLP1 or motility initiates host Ca^{2+} signaling remains to be determined.
We have added temporal indicators of the model, which is now Fig 8.

4. Fig2A label on the x axis should indicate ionomycin not zaprinast
This has been corrected.

5. Figure S2 is empty.
This supplemental figure included an animated gif image. It is necessary to enable viewing a gif animation in Adobe Acrobat, which might have been an issue.
We checked the pdf file prior to resubmission and the gif animation was working as expected.

6. Figure 3F/3G: how do the authors explain the delay in calcium in the plp1? Why is calcium level higher (as observed with pH) in the mutant?
The starting RGECO intensity (in arbitrary units) is variable from one PV to another, even for the same strain. Since we did not normalize for the size of the PV, larger PVs start with higher signal. Also, RGECO is not ratiometric, thus the readout indicates relative changes in Ca^{2+} levels for a single PV. We have included new examples that better represent most of the patterns observed. We did not detect a difference between strains in the initial elevation of parasite Ca^{2+} that occurs shortly after induction.

7. Figure 5F and 5H: it would be useful to show triple and double mutants in the same graph, to allow direct comparison.

Figures 5F and 5H have been combined into Fig 5F.

Other changes

1. We expanded the description of a general model for the activation of egress in the introduction.
2. We changed references to figures to conform with PLOS Pathogens format(e.g., Fig 1, S1 Fig, S1 Video, etc).
3. We added supplemental videos of pseudocolored ratios for PV pH corresponding to the examples shown in Figs 1D and 1G.
4. We moved the paragraph describing our attempts to visualize the directionality of PV acidification (PV before host cytosol or host cytosol before PV) to a place later in the results section for better flow.
5. For Fig 1C, we removed the thin connecting line and changed the error bars so that they extend above and below the data points, to be consistent with other graphs.
6. We changed some of the font sizes on the graphs to make the text more visible.
